# Circadian rhythms in haematological malignancies: therapeutic potential and personalised interventions

**DOI:** 10.1016/j.ebiom.2024.105451

**Published:** 2024-11-19

**Authors:** Marjan Motiei, Raed Abu-Dawud, Angela Relógio, Chalid Assaf

**Affiliations:** a Institute for Molecular Medicine, MSH Medical School Hamburg, Hamburg 20457, Germany; b Institute for Systems Medicine, and Faculty of Human Medicine, MSH Medical School Hamburg, Hamburg 20457, Germany; c Department of Dermatology, HELIOS Klinikum Krefeld, Krefeld 47805, Germany

**Keywords:** Circadian rhythms, Non-Hodgkin lymphoma, Cutaneous lymphoma, CTCL, Leukaemia, Therapeutic strategies, Chronotherapy

## Abstract

The circadian clock, a fundamental cellular mechanism, regulates the rhythmic expression of numerous genes and biological processes across various organs. Disruptions in this system, driven by genetic or environmental factors, have been reported to be involved in cancer progression. This review explores the role of the circadian clock in cancer hallmarks and its impact on cellular homeostasis within haematological malignancies. Drawing on findings from *in vitro*, *in vivo*, and clinical trials, this review highlights the potential of clock genes as diagnostic and prognostic biomarkers, and as therapeutic targets for optimising treatment timing. It discusses how circadian rhythms can enhance treatment efficacy through both pharmacological and non-pharmacological interventions, outlining strategies for optimising dosing schedules and implementing personalised chronobiological interventions, with a particular focus on haematological malignancies, including cutaneous lymphoma. Ongoing research holds promise for advancing personalised therapeutic approaches and ultimately improving cancer care standards.

## Introduction

Haematological malignancies (HM; for abbreviations see Supplementary Table) represent a significant global health burden, consisting of leukaemia and malignant lymphoma, including Hodgkin lymphoma (HL) and Non-Hodgkin-Lymphoma (NHL). Since 1990, the incidence of both leukaemia and NHL has steadily increased. In 2019, the age-standardised death rates (ASDR) for leukaemia and NHL were 4.26 and 3.19 per 100,000 population, respectively.^1^ According to GLOBOCAN 2022, there were approximately 487,000 new cases and 305,000 deaths globally for leukaemia, and 553,000 new cases and 250,000 deaths for NHL.^2^ Developed countries show higher incidence rates across the sexes, with mortality rates comparable and a slight male predominance.^2^ The burden of these cancers varies by sex, age, and region, with men generally more affected.^1^ Leukaemia is characterised by rapid abnormal blood cell production in the bone marrow and lymphatic system and is classified as acute or chronic and myeloid or lymphoid, depending on its cellular origin.^3^ Cutaneous lymphoma (CL), a subset of NHL, primarily affects the skin and can spread to lymph nodes, bone marrow, and internal organs in advanced stages.^4^ CLs are further categorised into cutaneous T-cell lymphomas (CTCL) and cutaneous B-cell lymphomas (CBCL), each with distinct clinical, histological, and molecular characteristics.^5^

**Fig. 1:**
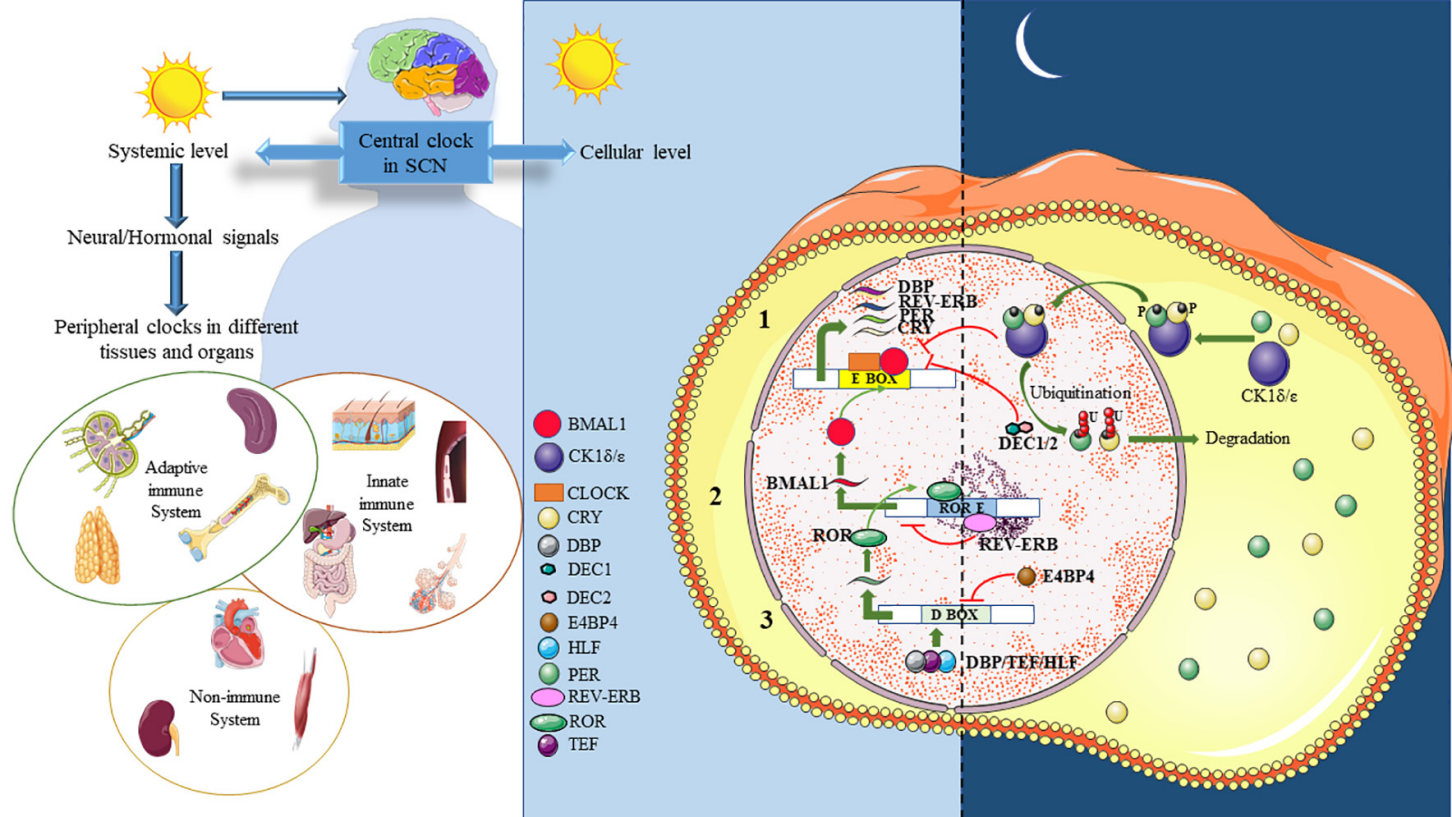
**Two hierarchical levels of the circadian system – systemic and cellular, including central and peripheral clocks.** At the systemic level, the central clock in the SCN governs the body's overall rhythm by responding to light cues, coordinating physiological processes through neural and hormonal signals to peripheral tissues. At the cellular level, the circadian system involves three interlinked transcription–translation feedback loops, including self-sustaining negative feedback loop (1), *REV-ERB/ROR* feedback loop (2) and protein regulation feedback loop (3). The autonomous peripheral clocks within cells regulate local processes, maintaining tissue and organ circadian rhythms and facilitating synchronised physiological functions.

Considering the distinct clinical characteristics of HMs, understanding circadian rhythms is crucial for uncovering disease mechanisms and developing new therapies. The circadian system, featuring a central clock in the suprachiasmatic nucleus (SCN) and peripheral clocks in various organs, including both immune (innate and adaptive) and non-immune systems (heart, kidneys, muscles), maintains homeostasis at both systemic and cellular levels^6^^,^^7^ (Fig. 1). Light, the primary “time cue” or “zeitgeber”, synchronises this system through intrinsically photosensitive ganglion cells (ipRGCs) in the retina, which signal the SCN.^8^ In contrast to *Drosophila*, in humans temperature acts as a zeitgeber for peripheral clocks but does not directly affect the central pacemaker in the SCN. Instead, the SCN uses body temperature to indirectly synchronise other clocks and maintain circadian harmony.^9^^,^^10^

The SCN coordinates circadian rhythms by sending signals to peripheral clocks through neurotransmitters, hormones (e.g., melatonin, glucocorticoids, catecholamines), metabolic factors, and body temperature.^8^ Melatonin influences clock genes such as *PER1* and *NPAS2*, especially in the SCN, binding to MT1/MT2 receptors to initiate signalling pathways that stabilise clock proteins like BMAL1, CRY, and PER through proteasome inhibition at night.^11^ Glucocorticoids reset peripheral clocks via the human glucocorticoid receptor alpha (hGRα), affecting clock genes like *Rev-ERBα* and *RORα*.^12^ Catecholamines influence *PER1* and clock gene oscillations through α1 and β2 adrenergic receptors.^13^ Peripheral clocks operate independently, respond to local cues, and provide feedback to the SCN through complex feedback loops involving endocrine signals and feeding cycles, indicating a coordinated system rather than simple top-down control.^14^

At the cellular level, the circadian system operates through three interlinked transcription–translation feedback loops involving key clock genes like *CLOCK*, *BMAL1*, *NPAS2*, *PERs*, *CRYs*, *RORs*, *REV-ERBs*, *DECs*, *CK1ε*, *NONO* and *TIM*.^15^ The first feedback loop begins during the day when CLOCK or NPAS2 forms heterodimers with BMAL1. These heterodimers bind to E-box elements and stimulate the expression of *PER1-3* and *CRY1-2* genes^16^ (Fig. 1). The proteins then accumulate in the cytoplasm and regulate rhythmic gene expression through E-box methylation and demethylation.^15^^,^^17^ In the late afternoon or evening, PER and CRY proteins form heterodimers, interact with casein kinase 1δ/ε (CK1δ/ε), and translocate to the nucleus to suppress CLOCK and BMAL1 activity^8^^,^^15^ (Fig. 1). Subsequently, PER and CRY proteins are targeted for degradation through polyubiquitination by E3 ligase complexes, including β-TrCP, which ubiquitinates PER, and FBXL3, which degrades CRY.^18^ DEC1 and DEC2 further inhibit *PER1* expression by competing with CLOCK for binding to BMAL1 or E-box elements. TIM regulates PER1 nuclear entry and its expression-suppression.^16^ This feedback loop eventually resets, allowing BMAL1/CLOCK activity to initiate a new cycle the next morning.

In the second feedback loop, *REV-ERB (α, β)* and *ROR (α, β, γ)* regulate *BMAL1* expression by binding to ROR/REV-ERB-response elements (RORE), with *RORs* activating and *REV-ERBs* inhibiting *BMAL1*^15^ (Fig. 1). *CLOCK* and *BMAL1*, in turn, activate the transcription of key circadian genes including *PERs, CRYs*, *REV-ERBs*, and *RORs*. The third feedback loop involves PAR-bZIP proteins (DBP, TEF, and HLF) and the repressor E4BP4 (NFIL3), which bind to D-boxes and are regulated by either the *BMAL1/CLOCK* or the *REV-ERB-ROR* loops^19^ (Fig. 1). These feedback mechanisms interact with E-box, RORE, and D-box elements in gene promoters and enhancers, maintaining a core feedback cycle of approximately 24 h.^16^

These feedback loops influence clock-controlled genes (CCGs) to regulate cellular functions and behaviours through mechanisms such as phosphorylation, acetylation, and ubiquitination, mediated by enzymes like kinases/phosphatases, acetyltransferases/deacetylases, and ligases/proteasomes.^15^^,^^20^ For instance, CLOCK phosphorylation by GSK-3β is crucial for its degradation and the maintenance of circadian rhythms. Similarly, PER1 protein levels fluctuate daily due to phosphorylation by CK1, affecting its degradation and nuclear entry.^21^ Additionally, epigenetic factors, including miRNAs and DNA methylation, modulate these loops, with specific miRNAs like miR-132/219, miR-192/194, miR-34, and miR-155 targeting *PER1*, *PER1/PER2*, *PER1*, and *BMAL1*, respectively.^22^

Understanding these circadian mechanisms is crucial for chronotherapy, which aligns treatment timing with the body’s natural cycles. Chronotherapy offers potential advantages such as enhanced treatment efficacy, reduced toxicity, and cost-effectiveness by optimising drug administration.^23^ It has demonstrated benefits in various cancers, including rectal cancer,^24^ glioblastoma,^25^ and metastatic renal cell carcinoma,^26^ by improving drug effectiveness and minimising side effects. However, chronotherapy faces challenges, including complex interactions with cancer rhythms, patient variability, and practical implementation issues.^27^ This paper explores how addressing circadian disruptions could revolutionise treatment paradigms and improve outcomes for patients with haematological malignancies.

## Clock genes in haematological malignancies

### Critical factors disrupting circadian rhythms and implications

Disrupted circadian rhythm significantly impact progression of HMs.^28^ Factors such as sleep disruptions, irregular eating habits, environmental pollutants, and oxidative stress all affect the circadian clock. Sleep issues from shift work, artificial light at night (ALAN), and psychosocial stress primarily disrupt the central clock in the SCN.^29^, ^30^, ^31^ Altered melatonin levels, influenced by ALAN or temperature changes, contribute to these disturbances.^32^ Increased outdoor ALAN is linked to a higher risk of NHL, especially diffuse large B-cell lymphoma (DLBCL).^30^ Shift workers also show greater circadian gene disruption in chronic lymphocytic leukaemia (CLL), particularly in *MYC*, *CCND1*, and melatonin, but no significant link to other B-cell lymphoma (BCL) subtypes.^29^^,^^32^

Eating disturbances also disrupt circadian rhythms, affecting intestinal microbiota and inflammation, which may contribute to cancer.^33^ In mice, irregular eating times disrupt circadian rhythms and colon activity, promoting colorectal cancer by reducing short-chain fatty acid–producing bacteria and inducing a proinflammatory environment.^34^ Meanwhile, fasting has been shown to inhibit the progression of B cell and T cell acute lymphoblastic leukaemia (B-ALL and T-ALL, respectively), but not acute myeloid leukaemia (AML).^35^ Environmental pollutants and oxidative stress can further disrupt circadian rhythms, influencing cancer development and treatment.^3^ Zhu et al. suggested that environmental disruptions to circadian rhythms may affect immune responses and NHL susceptibility.^36^

## Circadian rhythms and cancer hallmarks

**Fig. 2:**
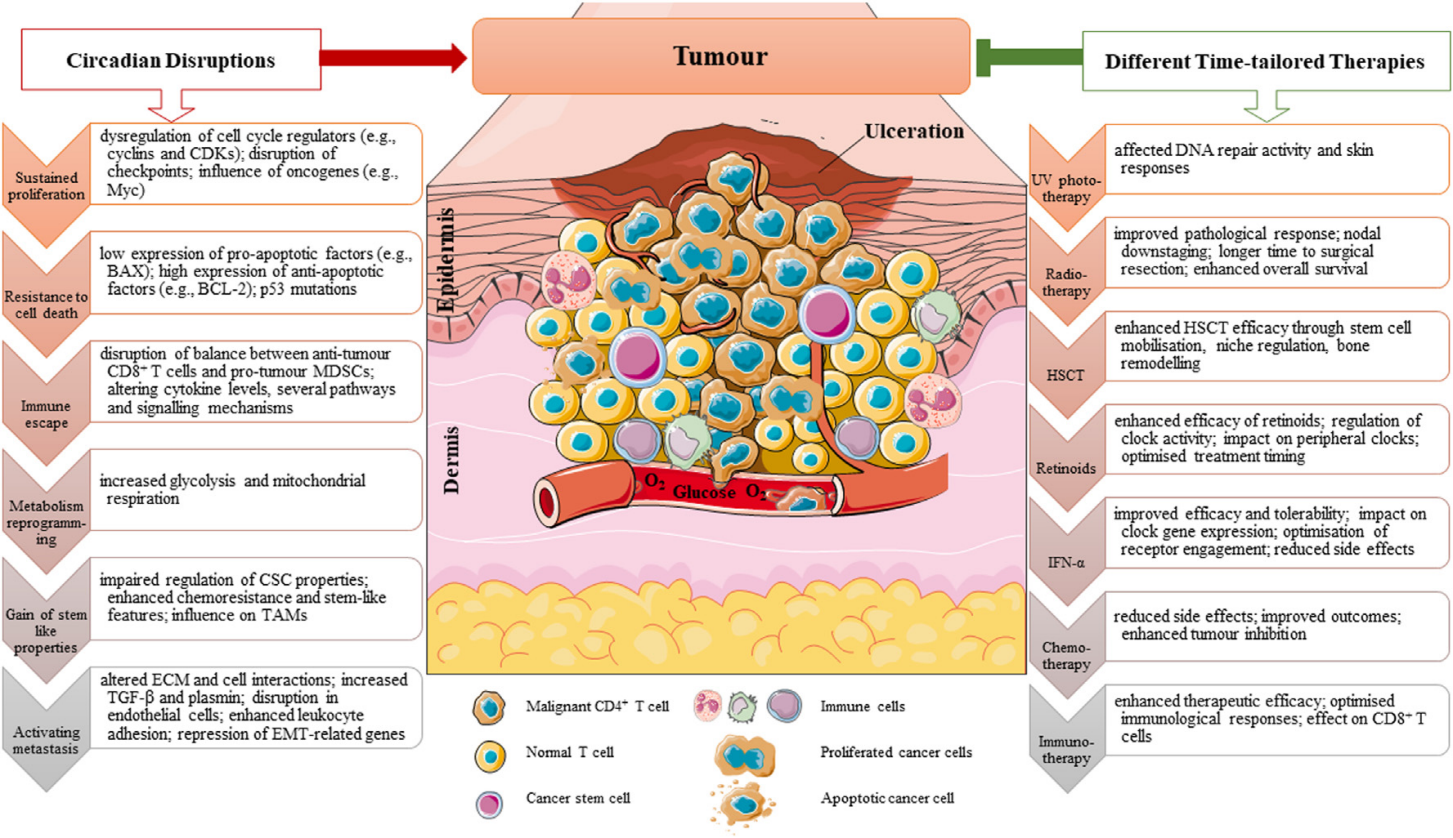
**Potential impact of circadian disruptions on tumour progression and tailored therapeutic approaches in haematological malignancies exampled in cutaneous T cell lymphoma**.

Disruptions in circadian rhythms significantly influence cancer development by affecting key signalling pathways associated with major cancer hallmarks, which are particularly relevant in haematologic malignancies like leukaemia and lymphoma (Fig. 2). Additionally, Table 1 summarises key studies that explore the role of circadian clock genes in these malignancies and their impact on disease progression.

**Table 1:** Summary of studies investigating the role of circadian clock genes in various haematologic malignancies.

Malignancy	Clock gene(s)	Role/impact on malignancy	Ref.
AML	*BMAL1*	Elevated by time-restricted feeding and low-sucrose diet.	^37^
T-ALL	*CLOCK, BMAL1*	Driving IL20R expression, prompting JAK/STAT signalling, and promoting leukaemia-initiating cell (LIC) activity	^38^
APL	*ARNTL*	Regulating cell viability and the cell cycle	^39^
CML	*PER2*	Regulating cell proliferation and cell cycle progression	^40^
CML	*BMAL1*	Influenced by therapeutic strategies that target SIRT1 activity	^28^
AML, ALL	*PER1-3, CRY1-2, BMAL1, TIM, CK1ε*	Altered expression of clock genes, notably *PER3* up-regulation, may be biomarkers for acute leukaemia progression and treatment response	^41^
CLL	*BMAL1, PER1-2*	Aberrant expression disrupts MYC and CCND1, affecting cell proliferation and apoptosis	^32^
ALL, AML, DLBCL	*BMAL1*	Silencing through hypermethylation disrupts circadian rhythms, with its reintroduction inhibiting tumour growth and its depletion promoting it.	^42^
BCL	*NPAS2*	Ala394Thr polymorphism linked to reduced NHL risk, suggesting certain circadian gene variants may act as tumour suppressors and potential biomarkers.	^43^
DLBCL	*PER2*	Regulated by C/EBPα; disruption indicates a significant tumour suppressive pathway	^44^
NHL	*CRY2*	Potential biomarker and key factor in NHL development	^45^
HL	BMAL1, CLOCK, PER, CRY	Interacting with TNFα, influencing cell proliferation and migration	^46^

### Sustained proliferation

**Fig. 3:**
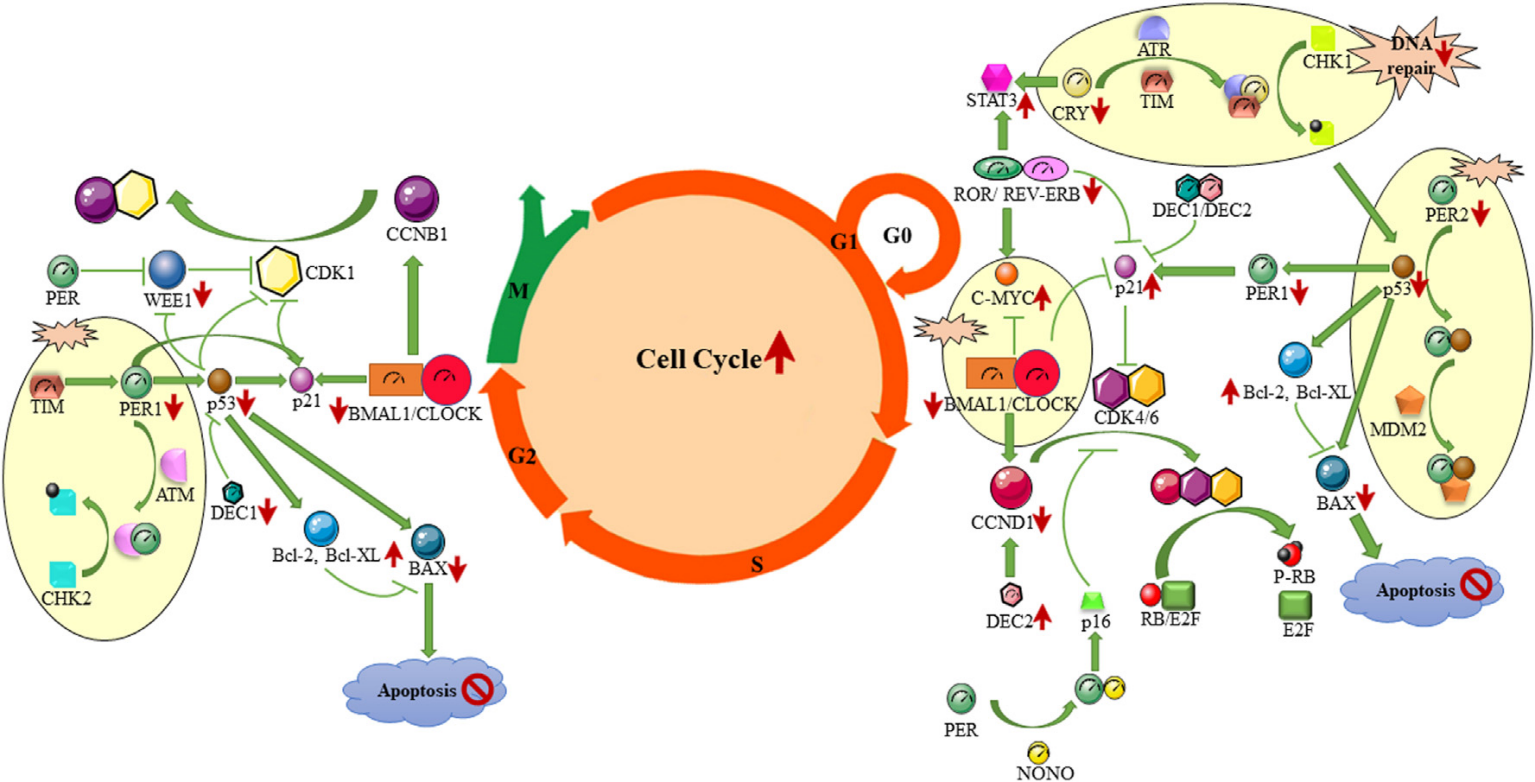
**Exploring the interplay between clock genes and cellular homeostasis, including cell cycle and DNA damage/repair, and apoptosis regulation.** Green arrows and T-shaped lines signify maintenance and regulatory pathways in a normal cell, while red arrows indicate up-regulation and down-regulation associated with tumour cell growth.

The BMAL1/CLOCK heterodimer regulates cell cycle rhythms by influencing cyclins (e.g., cyclin D1 (CCND1), cyclin B1 (CCNB1)), checkpoint kinases, and CDK inhibitors.^47^ During the G0/G1 transition, BMAL1/CLOCK activates *MYC*, which induces *CCND1* expression, facilitating G1 to S phase progression^48^ (Fig. 3). CCND1 interacts with CDK4/6, crucial for DNA synthesis and cell cycle progression.^49^ *BMAL1* also affects the G1 and G1/S checkpoints by downregulating *p21*, inhibiting cyclin–CDK complexes, and modulating *p53*.^48^ Additionally, BMAL1/CLOCK modulates *CCNB1* and *WEE1* to regulate the G2/M phase and promote mitotic entry.^48^

In *Bmal1/Clock* knockout mouse fibroblasts, cell division is delayed due to reduced *CCNB1*, downregulated *Wee1*, and upregulated *p21*.^48^ Similar *WEE1* reductions occur in lymphomas and leukaemia.^50^ *BMAL1* expression is notably reduced in large B-cell lymphoma (LBCL), CLL, and AML compared to healthy individuals.^20^ *BMAL1* disruption, via promoter hypermethylation or BMAL1/CLOCK axis dysregulation, increases *MYC* amplification and *CCND1* upregulation.^32^^,^^42^ *MYC*, essential for lymphocyte proliferation, is regulated by *E2A*, with overexpression and *TP53* mutations contributing to Sézary syndrome (SS).^51^ Abnormalities in *CCND1*, *RB1*, and *TP53* are seen in CTCL, affecting cell cycle regulation.^52^ NPAS2, with BMAL1, regulates the *MYC* promoter, influencing cell cycle dynamics and potentially affecting tumour growth. Genetic links associate *NPAS2* with higher risk of NHL, particularly BCL.^43^

PER proteins are also crucial for maintaining cellular homeostasis by regulating key cell cycle checkpoints, including G1/S and G2/M, through regulators such as CCND1, CCNB1, and CCNE1, which are essential for proper cell cycle progression^15^ (Fig. 3). They interact with NONO to control the *p16* promoter, influencing the G1/S transition.^48^ PER proteins also regulate p53 by binding to the *PER2* promoter and stabilising p53 against degradation by MDM2, thereby inhibiting cell cycle progression. MDM2, which promotes p53 degradation, also interacts with ARF, a tumour suppressor involved in *MYC*-mediated cell cycle checkpoints.^53^ At the G2/M checkpoint, *PER1* is critical for inhibiting *WEE1* kinase transcription, modulating the CCNB1–CDK1 complex, and influencing p53-dependent regulation of *WEE1* and *CCNB1*.^54^ Additionally, PER1 interacts with ATM and CHK2 to activate the *ATM* checkpoint pathway, ensuring proper G2/M progression.^55^ Disruption in PER protein function significantly contributes to tumourigenesis. For example, mutations in *Per2* in mice affect cell cycle regulation and tumour suppressor genes such as *Ccnd1*, *Cyclin A*, *Mdm2*, *Gadd45α*, and *c-Myc*.^56^ Low *PER2* expression, indicated by elevated CCND1 and CCNE levels, is observed in lymphoma cell lines and AML.^15^ Additionally, PER proteins impact *p21* expression, influencing *CDK2*, *CDK4*, and *CDK6* inhibition, which may contribute to alternative growth strategies like *CDK6* upregulation in SS.^51^

In addition, *CRYs* are crucial for cellular homeostasis, particularly at the G1/S transition. They inhibit proliferation by suppressing *WEE1* and modulating the DNA damage response^15^ (Fig. 3). Post-translationally, CRYs interact with TIM to regulate CHK1/ATR, important for G1/S transition.^48^ Disruption of *CRY* function impairs cell growth and DNA damage response, contributing to cancer. For example, CRY2, part of the SCFFBXL3 complex, promotes the ubiquitination of c-MYC, inhibiting growth in cancer cells.^57^ Furthermore, *CRY2* knockout increases DNA damage under stress, highlighting its role in maintaining cellular function.^58^

Similarly, *RORs* and *REV-ERBs* are essential for cell cycle regulation at the G1/S transition. They compete for the RORE element on the *p21* promoter, influencing cell cycle progression.^59^ *REV-ERBα* and *RORα* also affect *N-MYC* and *c-MYC* expression, crucial factors for cell cycle control and tumourigenesis.^60^ By regulating these factors, *RORs* and *REV-ERBs* maintain cellular homeostasis and can impact cancer development through their effects on cell cycle dynamics.

*DEC1* and *DEC2* are additional critical factors in regulating cell survival and proliferation by influencing key cell cycle genes such as *p21*, *p27*, *p57*, *c-MYC*, and *CDK* inhibitors.^15^ *DEC1* influences the G2/M checkpoint by modulating p53 and affects the DNA damage response through the inhibition of BMAL1/CLOCK activity.^49^ Elevated *DEC1* expression can enhance chemotherapy sensitivity in chronic myeloid leukaemia (CML) cells and provide protection against multidrug resistance (23). In contrast, high *DEC2* expression exhibits oncogenic properties and contributes to leukaemia progression.^61^

## Resistance to cell death

*BMAL1* is essential for apoptosis and cellular homeostasis by regulating pro-apoptotic (p53, BAX) and anti-apoptotic factors (BCL-2); its disruption impairs apoptosis and promotes tumour growth^48^ (Fig. 3). For instance, the BMAL1/CLOCK complex interacts with SIRT1, an NAD⁺-dependent deacetylase, to promote DNA repair and inhibit apoptosis particularly through the de-acetylation of proteins like p53 and Ku70.^62^ Additionally, *NPAS2* deficiency impacts circadian gene oscillation, such as *PER1*, which regulates apoptosis.^63^

In parallel, dysfunction in PER proteins contributes to cancer progression by affecting apoptosis. They stabilise p53, enhancing its tumour-suppressive role, and counteract inhibitors like MDM2^15^ (Fig. 3). In mice, *Per2* overexpression lowers anti-apoptotic factors (c-Myc and Bcl-2) and increases pro-apoptotic factors (p53 and Bax). Conversely, *Per2* deficiency disrupts tumour suppressors (*Ccnd1* and *c-Myc*), promoting tumour growth.^64^ In HL cells, *PER2* suppression reduces *BMAL1* and CLOCK/BMAL1 activity, leading to higher *c-MYC* and impaired apoptosis.^46^ *PER3*, however, promotes apoptosis by upregulating *Cleaved-CASP3* and *BAX*.^65^ Similarly, *DEC1* and *DEC2* also impact apoptosis, with *DEC1* promoting and *DEC2* inhibiting it.^66^ Overall, disruptions in molecular clock rhythms impair apoptosis, which may contribute to NHL progression by affecting cell death balance.^42^

### Activating metastasis

The extracellular matrix (ECM) and various cells, such as lymphocytes in the tumour microenvironment (TME), are crucial for cancer progression and metastasis. Factors like MMP9, MMP2, uPA, Cathepsins, Integrins, VEGF, and EMT transcription factors (e.g., SNAIL, SLUG, TWIST) drive cancer spread and secondary tumours.^67^ Circadian rhythms, especially *BMAL1* and *PER2*, influence these processes.^68^ In mice, reduced BMAL1 levels increase TGF-β and plasmin, promoting metastasis via the PAI-1-TGF-β-myoCAF pathway.^69^ In endothelial cells, *BMAL1* deletion disrupts migration-promoting molecules, while *CLOCK* overexpression enhances leukocyte adhesion.^70^ In HMs, *BMAL1* hypermethylation affects genes related to proliferation and survival, contributing to metastasis.^42^

Similarly, *PER2* affects cancer metastasis by repressing EMT-related genes like *TWIST1* and *SLUG* through interactions with polycomb proteins and HDAC2. Loss of PER2, or its degradation under hypoxia, increases EMT and invasion, leading to poorer prognosis and enhanced metastasis in breast cancer.^68^ Fu et al. found that *Per2*-deficient mice exhibit deregulation of tumour suppressor genes such as *Ccnd1* and *c-Myc*, impairing the DNA damage response and promoting tumour growth and metastasis.^56^ Abreu et al. reported that *shREV-ERBα* increased migration in HL cells, while *shTNF* and *shBMAL1* reduced it, highlighting the role of *CCN* genes and *TNF* in HL progression.^46^

### Immune escape

Circadian rhythms are crucial for regulating immune responses, particularly by balancing the activity of anti-tumour CD8^+^ T cells and pro-tumour myeloid-derived suppressor cells (MDSCs).^71^ CD8^+^ T cells target cancer cells, while MDSCs suppress immune responses and inflammation, reducing CD8^+^ T cell effectiveness.^72^ Disruptions in circadian rhythms can accelerate tumour growth by altering cytokine levels and immune functions. Key circadian genes like *BMAL1* play a significant role in these processes. *Bmal1* depletion in mice promotes an anti-inflammatory state, disrupts CD4^*+*^ and CD8^+^ T cell distribution in lymph nodes, and increases tumour growth by reducing CD8^+^ T cells in tumours.^73^ It also affects tumour-associated macrophages (TAMs), altering their cytokine production via HIF1α, ROS, and NRF2 pathways, leading to an increase in M2 TAMs, which further promotes tumour growth.^74^

Additionally, the circadian clock regulates *IL20R* expression, affecting JAK3/STAT3 signalling and IL-17 expression in T-ALL and CTCL.^38^ TNF influences immune pathways like T-cell receptor (TCR) and NF-κB in CTCL and HL, promoting proliferation by activating NF-κB and suppressing *PER2*.^46^ *CRY1* overexpression reduces inflammation by modulating NF-κB and cAMP/PKA pathways, while *CRY2* silencing may enhance immune escape in NHL via IL-6 and STAT3.^75^ *CRY1* and *CRY2* directly inhibit TNFα and IL-6, while *REV-ERBα* controls IL-6 induction.^76^

*REV-ERBα* regulates macrophage activity by inhibiting CCL2. Blockade of *REV-ERBα* increases CCL2 levels, resulting in higher immune cell accumulation and worse disease outcomes.^77^ *RORα* and *RORγ* are also critical for immune escape and cancer progression. *RORα*, activated by TGF-β and IL-6 via the STAT3 pathway, affects Th17 cell differentiation and impacts immune responses and tumourigenesis.^78^ It supports the function of liver-resident natural killer (LrNK) cell and Group 1 Innate Lymphoid Cells (ILC1), and its absence can lead to increased apoptosis and more aggressive tumour progression. *RORγ* boosts anti-tumour immunity by shaping Th17 and Tc17 cells, counteracting immune evasion.^79^ These circadian regulators influence immune responses and inflammation, highlighting potential new therapeutic targets.

### Metabolism reprogramming

Tumours reprogram their metabolism to ensure a steady nutrient supply by increasing aerobic glycolysis, lipogenesis, fatty acid oxidation, and methionine synthesis, all of which are influenced by circadian rhythms. Core clock genes like *CLOCK* and *BMAL1* are crucial for metabolic balance, with NAD⁺ playing a key role in integrating circadian rhythms with metabolism. SIRT1 is vital for the circadian transcription of *BMAL1*, *CRY1*, and *PER2*.^80^ In mice, SIRT1 regulates NAD⁺ levels by controlling NAMPT, an enzyme involved in NAD⁺ biosynthesis, creating a feedback loop where CLOCK, BMAL1, and SIRT1 bind to the NAMPT promoter in a circadian-dependent manner.^81^ Additionally, rhythmic O-GlcNAcylation also affects the stability of BMAL1 and CLOCK, impacting both circadian rhythms and glucose metabolism.^82^

In macrophages, *BMAL1* regulates glucose metabolism by influencing glucose uptake and its conversion into succinate, which drives IL-1β production, and by modulating PKM2, which enhances IL-1β expression. Consequently, *BMAL1* deficiency increases glycolysis and mitochondrial respiration, leading to heightened inflammation.^74^ Additionally, oncogenes such as *MYC* and *N-MYC* disrupt circadian rhythms by inducing *REV-ERBα*, which reduces *BMAL1* levels and rhythmic activity, thereby impacting tumour growth and metabolism.^83^

NPAS2, another circadian protein, reprograms glucose metabolism in hepatocellular carcinoma (HCC) by upregulating glycolysis-related genes and downregulating *PGC-1α*. Its regulation of *HIF-1α* and association with miR-199 b-5p further promote cancer growth, making *NPAS2* a key player in circadian metabolism regulation and a potential therapeutic target in HCC.^84^ *CRY2* also affects cancer risk and metabolism, with genetic variants associated with NHL affecting immune response and blood system development.^45^ These haematological malignancies, which exhibit circadian-influenced metabolic patterns, underscore the significance of circadian regulation in cancer prevention.^85^

### Gain of stem-like properties

Cancer stem cell (CSCs) play a crucial role in tumour initiation, growth, and recurrence due to their ability to self-renew and differentiate into various cancer cell types. Unlike normal stem cells, CSCs drive cancer progression and contribute to treatment resistance. In lymphomas, such as anaplastic large cell lymphoma (ALK + ALCL), *SOX2* and *OCT4* are essential for maintaining the stem-like properties and chemoresistance of these cells.^86^ Similarly, in B-cell precursor acute lymphoblastic leukaemia (BCP-ALL), interaction with bone marrow mesenchymal stromal cells (BM-MSCs) enhances chemoresistance and promotes stem-like features.^87^ TAMs also increase CSC proportions through the PTN/β-catenin signalling pathway, further influencing CSC dynamics.^88^

Circadian clock genes, including *CLOCK* and *BMAL1*, are critical for regulating CSC properties and metabolic functions. Disruption of these genes can impair CSC viability and proliferation, as evidenced in AML, where altered *CLOCK* and *BMAL1* activity leads to enhanced differentiation and depletion of leukaemia stem cells (LSCs).^89^ In contrast, circadian regulators such as *PER2* and *PER3* generally act as tumour suppressors. Specifically, *PER3* reduces stem cell properties in prostate cancer stem cells (PCSCs) through the WNT/β-catenin signalling pathway, while PER2 affects glioblastoma stem cells (GSCs).^90^ These findings highlight circadian clock genes as potential targets for disrupting CSC function and improve treatment outcomes for haematological malignancies.

## Time-tailored therapy

### Time-tailored therapy: integrating circadian rhythms into cancer treatment

Chronotherapy aligns medication with the patient’s circadian rhythms, enhancing treatment efficacy, reducing toxicity, and improving quality of life. Unlike conventional cancer therapies, which often suffer from lack of specificity, leading to high costs and drug resistance, chronotherapy can be more cost-effective by minimising side effects and additional interventions. By optimising drug timing and metabolism, chronotherapy allows for personalised treatment and holds potential applications beyond cancer.^23^ However, developing effective cancer chronotherapy faces significant challenges, including the complex interactions between cancer and circadian rhythms, patient variability, and limited pharmaceutical incentives.^27^ Additional obstacles include identifying reliable circadian biomarkers, translating research into clinical practice, and addressing variability in circadian rhythms due to genetic, epigenetic, and lifestyle factors.^23^ Results from different clinical trials consistently show that targeting the circadian rhythm improves not only progression free survival (PFS) but also overall survival (OS).^91^ This indicates its enormous clinical value and potential to change clinical practice in the future (Table 2). Therefore, addressing the challenges of integrating circadian medicine into clinical practice will require focused strategies to exploit, target, and detect circadian rhythms.^96^

**Table 2:** Summary of chronotherapy applications and outcomes across cancer types.

Cancer type	Therapeutic approach and chronotherapy	Chronotherapy application	Key findings/outcomes	Ref.
Rectal cancer	Neoadjuvant Radiotherapy	After 12:00	Improved pathological response, nodal downstaging, and longer time to surgical resection.	^24^
Brain metastases	Radiotherapy	Morning/Afternoon	Morning treatment improved OS, particularly for females, patients with controlled primary disease, and those with breast cancer metastases.	^91^
High-grade gliomas	Radiotherapy	Before/After 12:00	No difference in progression-free survival or OS	^92^
Metastatic Renal Cell Carcinoma	IFN-α therapy	Afternoon	IFNAR2 downregulation within 48 h of IFN-α administration and recovery within another 48 h.	^26^
DLBCL	Chemotherapy with R-CHOP	Morning/Afternoon	Afternoon treatments improved efficacy and reduced toxicity, particularly in females.	^93^
Glioblastoma	Chemotherapy with TMZ	Morning/Evening	Morning treatments increased TMZ efficacy	^94^
Glioblastoma	Chemotherapy with TMZ	Morning/Evening	Morning treatments improved OS, particularly in patients with MGMT-methylation, and showed better long-term outcomes	^25^
Melanoma	Immune checkpoint inhibition with ipilimumab, nivolumab, pembrolizumab	Before/After 16:30	Before 16:30 showed higher overall survival.	^95^

Exploiting the circadian clock by timing drug administration to align with physiological rhythms enhances efficacy and reduces toxicity; for instance, temozolomide (TMZ) may be most effective when administered during peak *Bmal1* expression.^94^ Targeting the clock involves resynchronising disrupted rhythms with interventions like light therapy, time-restricted eating, and exercise.^97^^,^^98^ Other pharmacological strategies include stabilising clock proteins and developing small-molecule modulators of the circadian clock (SMMCCs), which can also alter circadian cycles and affect drug metabolism and outcomes.^96^ Examples include CLK8, which disrupts BMAL1/CLOCK interactions,^99^ and compounds like a derivative of 2-ethoxypropanoic acid and ARN5187, which target CRY1/2 and REV-ERBβ proteins,^100^ showing promise for treating circadian disorders and cancer.

Detecting the clock involves creating diagnostic tools based on circadian clock biomarkers to optimise treatment timing. Mutations and copy number variations in circadian clock genes, such as *ARNTL2* in colorectal cancer and *NPAS2*, *CLOCK*, and *PER3* in breast cancer, are linked to cancer progression, suggesting their potential as biomarkers for cancer risk and prognosis.^101^ Diagnostic tools such as the TimeTeller saliva test, DLMO test, and BodyTime assay evaluate circadian biomarkers, including cortisol, melatonin, and gene expression profiles related to circadian clock genes, to optimise treatment schedules. However, challenges remain, including issues with sampling timing, cost, and ongoing development.^102^ Incorporating these biomarkers into diagnostics can personalise therapies, improving efficacy and reducing side effects by aligning treatment with the body's circadian rhythms.

### Time-tailored therapy and its potential application in cutaneous lymphoma

CLs are managed with therapies tailored to the type and stage of disease, including skin-directed and systemic treatments.^103^ Skin-directed therapies, like topical agents and targeted radiation, address localised disease, while systemic therapies involve medications or immunotherapies that target cancer cells throughout the body.^23^ While these treatments are effective, the impact of circadian rhythms on both pharmacological and non-pharmacological therapies is an emerging and promising area of research. Given the lack of chronotherapy studies specific to CL, insights from chronotherapy in other HM could provide valuable strategies for optimising time-tailored therapeutic approaches. Table 2 summarises key studies on chronotherapy across various cancers, highlighting their potential to enhance therapeutic interventions in CL.

However, current therapies already used for a long time in the treatment in CL could be influenced by its effect on the circadian rhythm. As an example, UV phototherapy is a crucial nonpharmacological treatment for CL, affected by patient factors, disease characteristics, and timing.^104^ Research shows that higher morning *OGG1* activity enhances DNA repair from oxidative stress, which may reduce 8-Oxoguanine accumulation in healthy volunteers.^105^ Thus, timing UV phototherapy according to the circadian rhythm could improve outcomes by aligning treatment with peak DNA repair activity, especially in tumour cells with low repair capabilities. Addison et al. highlighted the need for time-tailored phototherapy regimens due to the interplay between circadian rhythms, UV exposure, and skin responses.^106^ Moreover, elderly patients, who may require stronger light exposure due to decreased responsiveness, could particularly benefit from such tailored approaches.^107^

Radiotherapy is another effective skin-directed therapy, particularly for lesions in sensitive areas like the face and genitalia.^23^ Despite its common use, chronoradiotherapy for CL remains underexplored. Table 2 indicates that timing can significantly impact outcomes in other cancers, such as rectal cancer and brain metastases.^24^^,^^91^ Investigating radiotherapy timing in CL could reveal similar benefits, potentially enhancing efficacy and reducing side effects.

For advanced CL, haematopoietic stem cell transplantation (HSCT) is the only curative treatment. Circadian rhythms influence HSC mobilisation, niche regulation, and bone remodelling.^108^ Sugiyama et al. demonstrated significant correlations between the expression of circadian clock genes (*CRY1*, *PER3*, *CRY2*, and *BMAL1*) and plasma noradrenaline levels in HSCs, suggesting that time-tailored interventions could improve HSCT efficacy.^109^ Combining nonpharmacological interventions with chronotherapy might provide a new approach to combat tumour progression and reduce side effects of traditional treatments, potentially enhancing outcomes in CL.

In pharmacological treatments, targeting clock genes and understanding their role within the cell cycle emerge as promising therapeutic strategies.^20^ Retinoids, both topical and systemic, manage cutaneous lesions in patients with CL.^23^ These compounds, including vitamin A, regulate clock activity, influencing clock gene expression and antioxidant levels, and influencing peripheral clocks like those in the hippocampus.^110^ Retinoic acid, for example, can alter *PER2* gene expression timing, highlighting the potential for hormonal signals to regulate peripheral circadian rhythms.^111^ Utilising this time-based regulation could optimise retinoid therapy, enhancing efficacy and minimising side effects.

Recombinant IFN-α, a key treatment for CL, interacts with circadian rhythms by affecting clock gene expression and light-induced gene activation in the SCN.^112^ Adjusting IFN-α dosing to align with circadian rhythms could improve therapy tolerability and effectiveness. As shown in Table 2, IFNAR2 expression in peripheral blood mononuclear cells peaks at night, suggesting that night-time administration of IFN-α could optimise receptor engagement and improve therapeutic outcomes.^26^ Timing IFN-α therapy with these circadian fluctuations may enhance efficacy and reduce side effects.

Chemotherapy, a non-selective pharmacological treatment, often damages healthy cells. However, chronochemotherapy, timing chemotherapy in align with the circadian rhythms, has shown promise in reducing side effects and improving outcomes. For instance, female patients with DLBCL receiving R-CHOP in the afternoon exhibited better outcomes, likely due to sex-specific daily fluctuations in leukocytes and neutrophils.^93^ Additionally, combining chronochemotherapy with photodynamic therapy in a murine model demonstrated that aligning treatment with circadian rhythms significantly inhibited tumour growth, suggesting potential benefits for humans.^113^

Circadian rhythms impact immunological responses, crucial for optimising immunotherapy.^114^ The timing of antibody administration affects CD8^+^ T cell numbers, phenotype, and PD-1 levels, with early-day immune checkpoint inhibition potentially improving survival in women.^115^ Additionally, targeting *ARNTL2* with immune checkpoint inhibitors could offer a novel anti-tumour strategy.^116^ These findings highlight the need to consider circadian rhythms in developing antibody-based therapies for CL and suggest further research in this area.

## *In vivo* models for studying cycle reversal, gene manipulation, and drug induction

Studies using day and night reversal models show that dim light at night (dLAN) disrupts circadian rhythms and delays re-entrainment in mice, especially with limited physical activity.^117^ Chronic dLAN exposure in aged female mice results in immune dysfunction, increased inflammation, and reduced lifespan, with males being less affected.^118^ Mammary tumours or their removal induce fatigue and neuroinflammation during the dark phase, highlighting the time-of-day effects on tumour-related changes.^119^ Tsinkalovsky et al. found that *Per2* exhibits circadian rhythms in stem cells, bone marrow, and liver cells, while *Per1*, *Rev-erbα*, and *Bmal1* show limited or no rhythmic expression in stem cells and bone marrow. Most clock genes in primitive haematopoietic stem cells do not display organised circadian rhythms, similar to rapidly proliferating organs, suggesting that bone marrow circadian clock gene expression is developmentally regulated.^120^

These findings underline the role of circadian clock genes in physiological regulation and emphasise the need to consider circadian factors in research and gene manipulation. For instance, PER2::LUC and PER1::LUC mice models show that circadian clocks in immune cells influence inflammatory responses.^121^ *BMAL1* in myeloid mice cells regulates miR-155 production, protecting against sepsis, while its deficiency disrupts circadian rhythms and immune responses.^122^ *Bmal1*-deficient mice and BV-2 microglial cells reduced pro-inflammatory cytokines and altered metabolism, illustrating *Bmal1*’s role in immune and metabolic regulation.^123^ The *Per2* gene affects resistance to endotoxic shock and inflammatory cytokine levels via natural killer (NK) cells and natural killer T (NKT) cells.^124^ Per2 mutant mice exhibit increased cancer susceptibility and altered tumour responses post-γ radiation.^56^ Additionally, the *Cry2* mutation (A260T) linked to familial advanced sleep phase (FASP) shortens circadian periods and advances sleep onset.^125^ *Rev-erbα* is essential for ILC3 function, with its absence impairing NKp46^+^ ILC3 development and cytokine production, illustrating the broad impact of circadian regulation on immune function.^126^

These insights into circadian gene functions highlight the utility of drug-inducible circadian rhythm models for studying and manipulating these rhythms. High-throughput screening has identified small molecules, such as CK1ε inhibitors and benzodiazepine derivatives, that modulate circadian periods and amplitudes.^127^ Small molecules targeting circadian regulators of mice like *Bmal1, Clock, Crys, and Rev-erbs* show promise for treating diseases like glioblastoma.^128^ Pharmaceutical agents like nobiletin and SR8278 enhance circadian rhythms and antiviral defences in mice.^129^ SR8278 reduces homocysteine levels, and methamphetamine injections shift circadian gene expression outside the mice SCN.^130^ Overall, drug-inducible models are crucial for advancing circadian biology and developing new therapies.

## Conclusion and outstanding questions

This review highlights the profound impact of circadian rhythm disruptions on haematological malignancies, particularly cutaneous lymphoma. These disruptions, influenced by environmental, genetic, and epigenetic factors, play a crucial role in cancer progression by altering key signalling pathways. While evidence strongly supports the benefits of maintaining a healthy circadian clock, including tumour suppression, enhanced therapy responsiveness, prolonged survival, and improved quality of life, the specific role of clock genes in cutaneous lymphoma remains underexplored.

Several outstanding unanswered questions emerge from this review: How do circadian rhythms regulate immune responses in the skin, and how might these rhythms influence the timing of biopsies and treatments? Additionally, clock genes hold promise as diagnostic biomarkers, yet there is a pressing need for non-invasive circadian measurement methods and for more accurate diagnostic tools. How can we improve the accuracy and practical application of these tools to incorporate reliably clock gene information for managing cutaneous lymphoma?

Customising treatment through circadian system resetting offers potential for enhancing therapeutic efficacy and extending patient survival. However, gaps remain in our understanding of circadian rhythm complexities, such as differences between cancerous and normal skin cells, the tumour microenvironment, and tissue-specific circadian variability. How can we address these variations to personalise and optimise chronotherapy?

Despite substantial clinical evidence, chronotherapy remains underutilised by physicians. What further research is needed to integrate chronotherapy effectively into cancer treatment protocols, and what evidence is required to validate its use as a standard adjuvant therapy? Moreover, how can clock genes be utilised for long-term monitoring of patient responses and relapse risks, and what are the practical implications of such monitoring?

Finally, the journey toward fully harnessing the potential of clock genes in managing haematological malignancies continues. Future directions must explore optimal dosing times, consider individual circadian differences, and address the personalisation complexities posed by factors such as chronotypes, epigenetic changes, age, sex, lifestyle, underlying diseases, and treatments. Collaborative efforts, advanced technologies and well-designed clinical studies are vital for translating circadian research into clinical practice. As we advance, the integration of chronobiological insights promises to revolutionise the diagnosis, prognosis, and treatment of haematological malignancies, including cutaneous lymphoma, shaping a new era in cancer care.

**Search strategy and selection criteria**

The literature search was performed on PubMed using the following search terms: (haematological malignancies), (lymphoma), (leukaemia), (cutaneous lymphomas), (cutaneous lymphoma treatment), (circadian clock genes cancer), (chronotherapy), (chronopharmacotherapy), (chronochemotherapy), and (chronoradiotherapy). These abstracts were screened by one specialist in lymphoma, one expert in circadian clock genes, and one independent reviewer. This collaborative approach helped minimize bias, and any discrepancies were resolved through discussion among the reviewers. Only articles published in English between 2006 and 2024 were included.

## Contributors

M.M.: literature review, analysis, visualisation and figure design, writing - original draft, writing review & editing, R.A.: writing review & editing; A.R.: writing review & editing, C.A.: conceptualisation, supervision, funding acquisition, writing - review & editing. All authors approved the final version of this manuscript.

## Declaration of interests

There are no conflicts of interest, and Funding sources had no role in the study design, interpretation, or writing of the report.
