# Supplementary material for: Circadian rhythms in haematological malignancies: therapeutic potential and personalised interventions
Source: eBioMedicine. 2024 Nov 19;110:105451. doi: 10.1016/j.ebiom.2024.105451 (PMC11617894; doi:10.1016/j.ebiom.2024.105451)
Supplement: Supplementary Table [file mmc1.docx]

| **Table S1. List of Abbreviations and Definitions** | |
| --- | --- |
| ALAN | Artificial light at night |
| ALK+ ALCL | Anaplastic large cell lymphoma |
| ALL | Acute lymphocytic leukaemia |
| AML | Acute myeloid leukaemia |
| APL | Acute promyelocyte leukaemia |
| ASDR | Age-standardised death rates |
| B-ALL | B-cell acute lymphoblastic leukaemia |
| BCL | B-cell lymphoma |
| BCP | B cell precursor |
| BM-MSCs | Bone marrow mesenchymal stromal cells |
| CBCL | Cutaneous B-Cell Lymphomas |
| CCGs | Clock-controlled genes |
| CCNB1 | Cyclin B1 |
| CCND1 | Cyclin D1 |
| CK1δ/ε | Casein kinase 1δ/ε |
| CL | Cutaneous lymphoma |
| CLL | Chronic lymphocytic leukaemia |
| CML | Chronic myeloid leukaemia |
| CTCL | Cutaneous T-cell lymphomas |
| CSC | Cancer stem cell |
| dLAN | dim light at night |
| DLBCL | Diffuse large B-cell lymphoma |
| ECM | Extracellular matrix |
| FASP | Familial advanced sleep phase |
| GSCs | Glioblastoma stem cells |
| hGRα | Human glucocorticoid receptor alpha |
| HL | Hodgkin lymphoma |
| HM | Hematologic malignancy |
| HSCT | Hematopoietic stem cell transplantation |
| ILC1 | Group 1 Innate lymphoid cells |
| ipRGCs | Intrinsically photosensitive ganglion cells |
| LBCL | Large B-cell lymphoma |
| LSCs | Leukaemia stem cells |
| LrNK | Liver-resident natural killer cell |
| MDSCs | Myeloid-derived suppressor cells |
| NHL | Non-Hodgkin lymphomas |
| **NK** | **Natural Killer cells** |
| **NKT** | **Natural Killer T cells** |
| OS | Overall survival |
| PCSCs | Prostate cancer stem cells |
| RORE | ROR/REV-ERB-response elements |
| **SAD** | **Seasonal affective disorder** |
| SCN | Suprachiasmatic nucleus |
| SMMCC | Small-molecule modulators of the circadian clock |
| SS | Sézary syndrome |
| T-ALL | T-cell acute lymphoblastic leukaemia |
| TAMs | Tumour-associated macrophages |
| TME | Tumour microenvironment |
| TMZ | Temozolomide |
